# Shuang-Huang-Lian prevents basophilic granulocyte activation to suppress Th2 immunity

**DOI:** 10.1186/s12906-017-2071-y

**Published:** 2018-01-03

**Authors:** Qiaoling Fei, Yixin Han, Ruijuan Qi, Yuan Gao, Lei Fang, Rui Hou, Runlan Cai, Yun Qi

**Affiliations:** 1 Institute of Medicinal Plant Development, Chinese Academy of Medical Sciences & Peking Union Medical College, Beijing, 100193 China; 2 Research Center for Pharmacology and Toxicology, Institute of Medicinal Plant Development, Chinese Academy of Medical Science and Peking Union Medical College, 151 North Ma Lian Wa Road, Haidian District, Beijing, People’s Republic of China

**Keywords:** Basophilic granulocytes (BGs), Shuang-Huang-Lian (SHL), Th2 immunity, IL-4, NFAT, Cytosolic Ca^2+^ (Ca^2+^_[c]_)

## Abstract

**Background:**

Basophilic granulocytes (BGs) not only initiate the induction of Th2 cell differentiation, but also amplify the ongoing Th2 response. Shuang-Huang-Lian (SHL) is clinically used for relieving type I hypersensitivity by continuous treatment for several weeks.

**Methods:**

ELISA, flow cytometry, magnetic activated cell sorting, isoelectric precipitation, hybridoma technique, transfection and luciferase reporter assay were used in this study. The statistical analysis was performed using a one-way ANOVA.

**Results:**

Our recently published study demonstrated that SHL exerted a remarkable effect on mast cell stabilization. Herein, we sought to elucidate the effect of SHL on shrimp tropomyosin (ST)-induced Th2 immunity and its underlying mechanisms. The obtained data showed that continuous treatment with SHL significantly suppressed ST-stimulated Th2-cytokines release and IgE synthesis. A mechanistic study indicated that SHL not only reduced BG early IL-4 release before ST-specific IgE (sIgE) production, but also inhibited BG activation in the presence of sIgE, including suppressing CD200R surface expression and decreasing IL-4 production. Moreover, SHL markedly decreased the cytosolic Ca^2+^ (Ca^2+^_[c]_) level and inhibited the nuclear factor of activated T cells (NFAT) activation in RBL-2H3 cells.

**Conclusions:**

Collectively, SHL potently reduces ST-induced Th2 immunity by inhibiting the BG Ca^2+^-NFAT pathway and, thus, suppressing the early IL-4 release before sIgE synthesis and inhibiting BG activation in the presence of sIgE. This study provides the pharmacological basis for the clinical use of SHL to relieve type I hypersensitivity by a successive dose regimen.

**Electronic supplementary material:**

The online version of this article (doi: 10.1186/s12906-017-2071-y) contains supplementary material, which is available to authorized users.

## Background

Naïve CD4+ T (NCT) cells, also known as T-helper (Th) cells, play critical roles in orchestrating adaptive immune responses to a variety of pathogens. They are also involved in autoimmunity, asthma, and allergic responses as well as in tumor immunity. NCT cells produced in the thymus migrate to the periphery where they encounter antigens. In different cytokine milieus, NCT cells differentiate into distinct subsets of effector cells, which allow for tailored immune responses against specific antigens. Th1 and Th2 cells are two major CD4 + effector T-cell subsets [1]. The Th1 cell immune response is characterized by the production of cytokines (e.g., IFN-γ), and the enhanced productions of IgG_2a_ and IgG_2b_ in mice. The Th2 response is characterized by the predominant production of IL-4, IL-5, IL-10, and immunoglobulins, mainly IgE [2, 3]. In addition to suppressing type 1 immunity and type 1-driven inflammation, type 2 immunity is a major effector response that has many important host-protective and pathogenic activities. One of the most important protective functions of type 2 immunity for host defense is to promote resistance to large extracellular helminth parasites, but it is detrimental in allergic diseases in humans [4]. Thus, the suppression of type 2 responses is a useful therapeutic strategy for the treatment of allergic diseases.

Unlike Th1 immunity, in which dendritic cells (DCs) present antigens and produce Th1-inducing cytokines, such as IFN-γ (and, in humans, also by type I IFN [5]), DCs fail to produce IL-4, the key driver of CD4+ Th2 cell responses. Therefore, the induction of Th2 responses may require an alternative (non-DC) antigen-presenting cell (APC). Under homeostatic conditions, mature basophilic granulocytes (BGs) are found circulating in the peripheral blood as well as in highly vascularized organs. However, upon immunological stimulation, the activated BGs rapidly migrate into draining lymph nodes (LNs) from the site of antigen injection or helminth infection and act as APCs by uptaking and processing antigens [6–10]. Indeed, many studies have suggested that BG-derived IL-4 is crucial for promoting Th2 skewing upon cysteine proteases, allergens and extracellular parasites [9, 11], and thus, BGs trump DCs as APCs for Th2 responses [12].

Shuang-Huang-Lian (SHL), a formula containing *Lonicerae Japonicae Flos*, *Scutellaria baicalensis* and *Fructus Forsythiae*, is consistently prepared by a stringent manufacturing procedure from Chinese Pharmacopoeia [13]. Clinically, SHL products are generally considered antimicrobial agents and are delivered through different routes (e.g., oral, intravenous and pulmonary routes, etc.) [13]. Our previous studies showed that SHL protects lung tissue from infections via its potential anti-inflammatory and anti-oxidative activities [14, 15]. Moreover, our more recent study revealed that a single intraperitoneal treatment of an SHL injection potently dampens compound 48/80-MrgprX2 and IgE-FcεRI mediated mast cell (MC) degranulation [16]. Nevertheless, SHL is commonly used for relieving type I hypersensitivity by a continuous treatment for several weeks [17, 18], suggesting that the function of SHL might not only be attributed to short-term MC stabilization, but also involve a long-term therapeutic effect. Thus, the present study focused on the effect of SHL on shrimp tropomyosin (ST)-induced Th2 immunity and its underlying mechanisms.

## Methods

### Materials

The SHL injection, prepared according to the Chinese Pharmacopoeia [13], was provided by Duoduo Pharmaceutical Co., Ltd. (Jiamusi, Heilongjiang, China). Pluonic F-127 and active papain were from Sigma-Aldrich (St Louis, MO, USA). Fluo-3 AM Ester was from Biotium (San Francisco, CA, USA). Mouse total IgE (tIgE), IL-4, IL-5 and IL-10 ELISA kits were from Biolegend Co. (San Diego, CA, USA). Mouse IL-13 and IFN-γ ELISA kits were from Excell Bio. Co. (Shanghai, China). Recombinant mouse IL-3 was from Novoprotein (Summit, NJ, USA). Mouse direct lineage cell depletion kit, CD3-FITC antibody, CD117-APC antibody, CD19-VioBright (TM) FITC antibody, CD117 Microbeads, FcεRI-PE antibody, anti-PE Microbeads and Red Blood Cell Lysis solution were from Miltenyi Biotec Inc. (Auburn, CA, USA). Protein G PLUS-Agarose was from Santa Cruz Biotechnology, Inc. (Santa Cruz, CA, USA). The HRP-labeled rat anti-mouse IgE antibody and HRP-labeled goat anti-rabbit antibody were from Abcam Co. (Cambridge, UK) and Jackson ImmunoResearch Laboratories Inc. (Lancaster, PA, USA), respectively. The rabbit anti-mouse IgG_2a_ and IgG_2b_ antibodies were from OriGene Technologies, Inc. (Rockville, MD, USA). The nuclear factor of activated T cells (NFAT)-luc plasmid and its control were synthesized by GenePharma Co., Ltd. (Shanghai, China). The FITC-labeled anti-mouse IgE antibody and PE-labeled anti-mouse CD200R antibody were from BD Biosciences (Franklin Lakes, NJ, USA) and eBioscience (San Diego, CA, USA), respectively. The luciferase assay system was from Beyotime Institute of Biotechnology (Haimen, Jiangsu, China). Imject alum adjuvant was from Thermo Fisher Scientific (New York, NY, USA). The other reagents were of analytical grade.

### Cell line and animals

The rat basophilic leukemia cell line (RBL-2H3) was obtained from the cell bank of the Chinese Academy of Sciences (Shanghai, China). The Balb/c mice (male, 18–20 g) were from Vital River Experimental Animal Services (Beijing, China) and housed in a SPF laboratory in standard temperature and humidity conditions with a 12 h light/dark cycle. They were randomly divided into 4 groups (8 mice per group): normal saline group (NS), ST model group and SHL groups (3 and 6 mL/kg). All the animal experiments were carried out according to the National Institutes of Health Guide for Care and Use of Laboratory Animals and approved by the Institutional Animal Care and Use Committee (IACUC), Institute of Medicinal Plant Development (IMPLAD) of Chinese Academy of Medical Sciences (Licence nos. 20160314, 20160426, 20160505). Anesthetic drugs and all other necessary measures were used to reduce animal suffering during experimental procedures.

### Isolation of ST and production of ST-specific IgE (sIgE) monoclonal antibody (mAb)

ST and ST-sIgE mAb were prepared as we previously described [14].

### Sensitization protocol

ST-sensitized mice were prepared as shown in Additional file 1: Figure S1. Mice were injected (i.p.) weekly (days 0, 7, 14 and 21) with imject alum containing ST (60 μg/mouse). Simultaneously, the mice were treated daily with SHL (3 mL/kg or 6 mL/kg, i.g.) or physiologic saline (normal control group and ST model group). 4 weeks later (day 28), the mice were anesthetized by inhalation of diethyl ether and euthanized by cervical dislocation. The blood, sera and spleens were collected for the experiments.

### Measurement of serum tIgE and ST-specific antibodies

Th1 cells regulate B cells to produce antigen-specific IgG_2a_ and IgG_2b_, while Th2 cells induce an allergic inflammation by promoting IgE class switching [2, 3]. To clarify the function of SHL on the Th1/Th2 phenotype, we evaluated the effect of SHL on ST-specific serum antibody production. The serum tIgE level was measured using a commercial mouse tIgE ELISA kit. The levels of sIgE were measured as previously described with some modifications [19]. Briefly, IgG in the serum was removed by Protein G PLUS-Agarose according to the manufacturer’s instructions. The 96-well microtiter plates were coated with ST (10 μg/mL, 100 μL/well) in coating buffer (0.05 M carbonate buffer, pH 9.6). After an overnight incubation at 4 °C, the plates were washed 4 times with PBS/0.05% Tween 20 and were blocked with 1% BSA-PBS at 37 °C for 1 h. After washing, the serum samples (1:100 dilutions) were added to the plates and were incubated overnight at 4 °C. After washing, 100 μL of the HRP-labeled rat anti-mouse IgE antibody (1:5000 dilutions) was added. The plates were incubated at 37 °C for 1 h. The reactions were developed with TMB for 5 min at 37 °C and were stopped by 100 μL of 2 M H_2_SO_4_. The optical density (OD) was read at 450 nm.

For measuring the ST-specific IgG (sIgG) in the serum, the serum samples (1:100 dilutions) were added in the plate coated with ST (10 μg/mL) followed by an incubation at 37 °C for 1 h. After washing, 100 μL of the rabbit anti-mouse IgG_2a_ or IgG_2b_ antibody was added. After a further incubation at 37 °C for 1 h and washing, 100 μL of the HRP-labeled goat anti-rabbit antibody (1:5000 dilutions) was added, and the plate was incubated at 37 °C for 1 h. The reactions were developed with TMB for 10 min at 37 °C and were terminated by 100 μL of 2 M H_2_SO_4_. The OD values were read at 450 nm. The serum ST-specific antibody levels were calculated by comparing the OD values.

### Splenocyte culture and cytokine measurement

In contrast to Th1 cells, which produce IFN-γ, Th2 cells produce cytokines, such as IL-4, IL-5, IL-10 and IL-13, which are important in switching antibody production from B cells to predominantly IgE synthesis against the allergen [20]. Thus, the effect of SHL on cytokine production in the splenocytes was assayed. Spleens from the ST-sensitized mice were taken and single cell suspensions were prepared aseptically in DMEM according to a previously described method [21]. The obtained splenocytes (4 × 10^6^ cells/well) were seeded in a 24-well plate and challenged by ST (8 μg/mL) at 37 °C for 72 h. The levels of IL-4, IL-5, IL-10, IL-13 and IFN-γ in the culture medium were measured using respective ELISA kits according to the manufacturer’s instructions.

### Measurement of cytosolic Ca^2+^ (Ca^2+^_[c]_) level

The Ca^2+^_[c]_ level in the RBL-2H3 cells was determined using the calcium-reactive fluorescence probe Fluo-3/AM as we previously described [16].

### BG activation test

#### Measurement of the IL-4 level produced by BG-rich splenocytes and sensitized RBL-2H3 cells

By producing Th2-promoting cytokines (e.g. IL-4), BGs promote Th2 skewing in response to various antigens in the absence/presence of DCs [7]. Thus, the effect of SHL on early IL-4 production in BG-rich splenocytes in response to papain or ST was investigated. BG-rich splenocytes were prepared as previously described with some modifications [10]. T cells, B cells and mast cells in spleen were depleted by a Direct Lineage Cell Depletion kit and CD117 Microbeads via a MACS (Miltenyi Biotec) negative selection (CD3^−^CD19^−^CD117^−^) and were then enriched via a MACS positive selection (FcεRI^+^). Briefly, the Balb/c mice ( n= 20) were anesthetized and sacrificed by cervical dislocation. The spleens were sterilely removed and dissociated by cell strainer to obtain single-cell suspension. The red blood cells from the spleen cell samples from the mice were removed by the Red Blood Cell Lysis solution. Next, 100 μL of Direct Lineage Cell Depletion Cocktail and 200 μL of CD117 Microbeads were added into the obtained cells (1 × 10^8^ cells). Mix well and incubate for 15 min at 4 °C. 6 mL of medium was added and the cell suspension was centrifuged at 300×g for 10 min at 4 °C. Aspirate supernatant completely and resuspend the cells by 1 mL medium. Prepare the LS column by rinsing with 3 mL of the medium and apply the cell suspension onto the column. Collect the unlabeled cell suspension that pass through and combine with the flow-through and centrifuge at 300×g for 10 min at 4 °C. The obtained cells were stained by 10 μL of anti-FcεRIα-PE antibody and 100 μL of anti-PE Microbeads for 15 min at 4 °C and centrifuged to aspirate supernatant. Apply the cell suspension onto the column and wash column with the 10 mL of medium. Pipette 3 mL of medium onto the column and immediately flush out the magnetically labeled cells (FcεRI^+^) by firmly pushing the plunger into the column. Collect the obtained cell suspension (FcεRI^+^). The purity of the BGs in the obtained cells was analyzed by using a FACSCalibur flow cytometer and the value was 13.66% (see Additional file 2: Figure S2). The obtained BG-rich splenocytes (1 × 10^6^ cells/well) were seeded in a 96-well plate and treated with SHL (0.5–2%) and ST (100 μg/mL) or active papain (100 μg/mL) in the presence of IL-3 (1 μg/mL) for 24 h. The supernatants were analyzed for IL-4 levels by ELISA.

To determine the effect of SHL on the BGs after sIgE synthesis, the RBL-2H3 cells were sensitized with anti-ST IgE (25 μg/mL) at 37 °C overnight. The cells were pretreated with or without SHL (0.5–2%) at 37 °C for 30 min and were then stimulated with ST (20 ng/mL) for 6 h. IL-4 levels in the supernatants were assayed by ELISA.

#### Measurement of the peripheral BG activation marker CD200R

BGs bind IgE through high affinity receptors (FcεRI) on their surface. Upon antigen stimulation, the activated BGs rapidly migrate into draining LNs and release a great amount of IL-4 to amplify Th2 responses [6, 9]. Thus, we determined the effect of SHL on CD200R surface expression, a marker of murine BG activation [22], in peripheral BGs from the ST-sensitized mice and IL-4 release in the anti-ST IgE-sensitized RBL-2H3 cells. For the CD200R assay, the whole blood from the ST-sensitized mice was collected in EDTA-K_2_ anticoagulative tube (100 μL/tube) and was incubated with ST at 5 μg/mL for 2 h at 37 °C. The cells were stained with a FITC-labeled anti-mouse IgE antibody and a PE-labeled anti-mouse CD200R antibody for 15 min at 25 °C. The erythrocytes were lysed, and the leukocytes were fixed using a whole blood lysing reagent kit for 5 min at room temperature. The cells were washed in 2 mL of PBS and resuspended in 300 μL of PBS for an analysis using a FACSCalibur flow cytometer [22].

### Plasmids, transfection and luciferase reporter assay

Given that the transcription of IL-4 is regulated by NFAT [23], we determined the effect of SHL on NFAT activation in the RBL-2H3 cells stably transfected with the pNFAT-luc plasmid. The RBL-2H3 cells were transfected with pNFAT-luc and its control plasmids by electroporation. Stable cells were obtained by G418 (400 μg/mL) selection. For the NFAT assay, the cells were sensitized by anti-ST IgE (25 μg/mL) overnight and were then pre-incubated with different concentrations of SHL, and 0.5 h later, the cells were stimulated by ST (20 ng/mL) for 6 h. The cells were lysed, and the luciferase activity was measured using the luciferase assay system.

### Statistical analysis

The data represent the mean ± SD of at least three independent experiments. The statistical analysis was performed using a one-way ANOVA. A student’s *t* test was used when only two groups were compared. The difference was considered statistically significant when *P* < 0.05.

## Results

### SHL decreases serum tIgE and sIgE levels in ST-sensitized mice

**Fig. 1:**
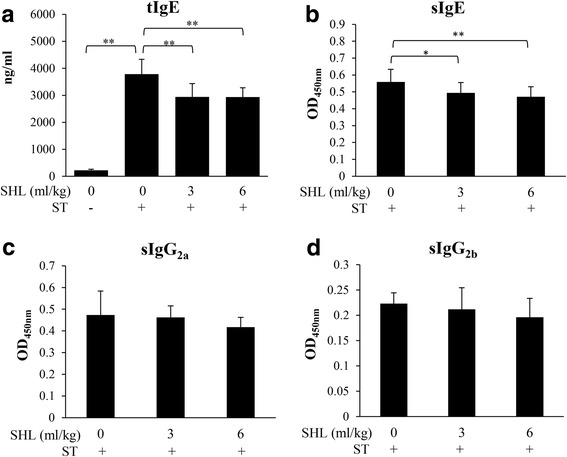
Effects of SHL on the serum tIgE (**a**), sIgE (**b**), sIgG_2a_ (**c**) and sIgG_2b_ (**d**) productions in the ST-sensitized mice. Mice were intraperitoneally immunized with ST (60 μg/mouse) once a week. SHL (3 mL/kg or 6 mL/kg, i.g.) was daily administrated daily to the mice. 4 weeks later the mice were sacrificed and the sera were collected. The serum tIgE was determined by a commercial ELISA kit. The sIgE, sIgG_2a_ and sIgG_2b_ levels were assayed by our established ELISA. The data represent the means ± SD (*n* = 10). *P < 0.05 and **P < 0.01

As shown in Fig. 1, ST induced sIgE, sIgG_2a_ and sIgG_2b_ production after 4 weeks of ST immunization, while SHL significantly reduced serum tIgE and sIgE levels (Fig. 1a-b and Additional file 3: Table S1) but did not affect sIgG_2a_ and sIgG_2b_ (Fig. 1c-d and Additional file 3: Table S1), indicating the suppressive effect of SHL on the ST-induced Th2 immunity.

### SHL suppresses Th2 cytokine production by splenocytes from the ST-sensitized mice

**Fig. 2:**
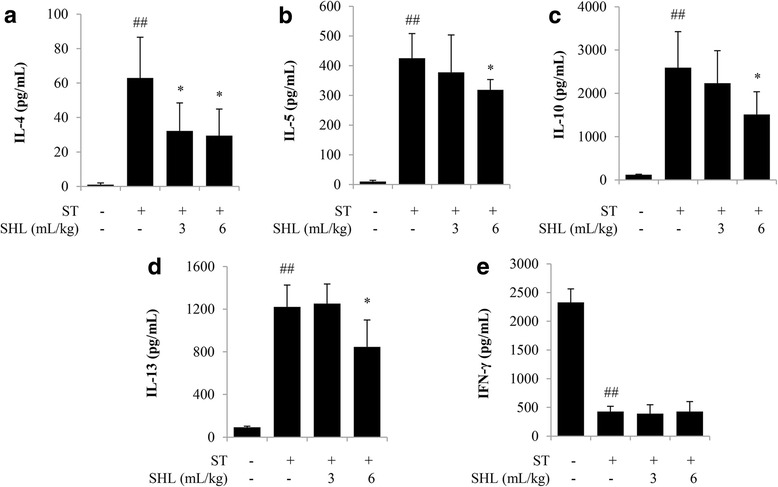
Effect of SHL on the cytokines produced by splenocytes. The mice were intraperitoneally immunized with ST (60 μg/mouse) once a week. SHL (3 mL/kg or 6 mL/kg, i.g.) was administered daily to the mice. 4 weeks later the spleens were taken, and single cell suspensions were aseptically prepared. The obtained splenocytes (4 × 10^6^ cells/well) were seeded in a 24-well plate and stimulated with ST (8 μg/mL) at 37 °C for 72 h. The levels of IL-4 (**a**), IL-5 (**b**), IL-10 (**c**), IL-13 (**d**) and IFN-γ (**e**), in the culture medium, were measured using the ELISA kits. The data represent the means ± SD (n = 10). ^##^*P* < 0.01 vs. the normal control group; *P < 0.05 vs. the ST group

As shown in Fig. 2, in vitro ST stimulation significantly increased the release of IL-4, IL-5, IL-10 and IL-13 but simultaneously decreased IFN-γ secretion. A continuous treatment with SHL (i.g.) significantly reduced ST-induced IL-4, IL-5, IL-10 and IL-13 productions (Fig. 2a-d and Additional file 4: Table S2) but did not affect IFN-γ release (Fig. 2e and Additional file 4: Table S2), indicating that SHL suppressed ST-induced Th2 immunity. Furthermore, splenocytes from the unsensitized normal mice showed no response to an ST challenge (data not shown).

### SHL suppresses BG activation

#### SHL decreases IL-4 release in BG-rich splenocytes upon papain or ST stimulus

**Fig. 3:**
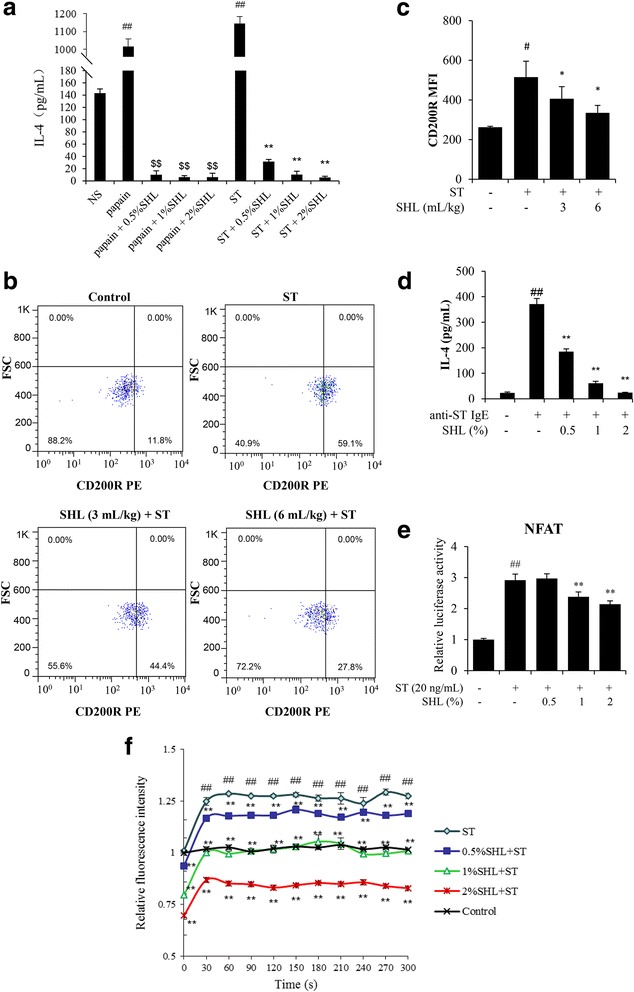
**a** SHL decreased early IL-4 produced by BG-rich splenocytes. Splenic BGs, preliminarily purified by a MACS system, were seeded in a 96-well plate (1 × 10^6^ cells/well) and treated with SHL (0.5–2%) and ST (100 μg/mL) or active papain (100 μg/mL) in the presence of IL-3 (1 μg/mL) for 24 h. IL-4 levels in the supernatants were analyzed by ELISA. ^##^*P* < 0.01 vs. NS; ^$$^*P* < 0.01 vs. papain alone; ^**^P < 0.01 vs. ST alone. **b-c** SHL inhibited BG CD200R surface expression. The whole blood from the ST-sensitized mice was collected and stained with anti-IgE FITC and anti-CD200R antibodies after an incubation with ST (5 μg/mL) for 2 h at 37 °C. BGs in the whole blood were identified by an anti-IgE FITC antibody. The surface expression of CD200R on the BGs was assayed by flow cytometry. Isotype control and unstained cells were used as the negative controls. **b** Representative dot plots of BG CD200R surface expression. **c** Effect of SHL on the mean fluorescence intensity (MFI) of CD200R in the BGs after stimulation with ST. **d** SHL decreased IL-4 release in the sensitized RBL-2H3 cells. The cells were sensitized with anti-ST IgE (25 μg/mL) at 37 °C overnight. The cells were pretreated with or without SHL at 37 °C for 30 min and were then stimulated with ST (20 ng/mL) for 6 h. IL-4 levels in the supernatants were assayed using a commercial ELISA kit. **e** SHL suppressed ST-induced NFAT activation in BGs. RBL-2H3 cells stably transfected with the pNFAT-luc plasmid were sensitized with anti-ST IgE (25 μg/mL) at 37 °C overnight. The cells were pretreated with or without SHL at 37 °C for 30 min and were then stimulated with ST (20 ng/mL) for 6 h. The cells were lysed and the luciferase activity was measured using the luciferase assay system. **f** SHL reduced Ca^2+^_[c]_ levels in the RBL-2H3 cells. The RBL-2H3 cells were sensitized with anti-ST IgE (25 μg/mL) at 37 °C overnight. The cells were loaded with Fluo-3 AM (4 μM) at 30 °C for 30 min. The stained cells were treated with or without SHL for 30 min and were then exposed to ST (20 ng/mL). The fluorescent intensity (λ_ex_ 485 nm and λ_em_ 538 nm) was recorded every 30 s. ^#^*P* < 0.05 and ^##^*P* < 0.01 vs. the control; ^*^P < 0.05, ^**^P < 0.01 vs. ST alone

As shown in Fig. 3a and Additional file 5: Table S3, papain markedly stimulated BG-rich splenocytes to release large amounts of IL-4. Intriguingly, ST also dramatically elevated IL-4 release. Whether papain or ST challenge, SHL potently reduced IL-4 production in a concentration-dependent manner without cytotoxicity (see Additional file 6: Figure S3).

#### SHL suppresses CD200R surface expression in peripheric BGs and IL-4 production in anti-ST IgE-sensitized RBL-2H3 cells

The obtained results (Fig. 3b-c and Additional file 5: Table S3) showed that in vitro ST stimulation resulted in a considerable increase in the percentage of CD200R surface expression (positive percentage = 59.1%) compared with that of the normal control group (positive percentage = 11.8%). Similarly, the mean fluorescence intensity (MFI) of the CD200R PE staining in the BG population also increased after ST application. However, SHL significantly suppressed BG CD200R surface expression with positive percentages of 44.4% (3 mL/kg) and 27.8% (6 mL/kg) and lower MFI values. In addition, an anti-FcεRI antibody only may bind to FcεRI that is not occupied by IgE. As a result, the antibody was not able to label the peripheral BGs in the ST-sensitized mice with high-level serum IgE. Hence, we used an anti-IgE antibody instead (Fig. 3b-c). Moreover, SHL potently decreased ST-induced IL-4 elevation concentration-dependently in the sensitized RBL-2H3 cells (Fig. 3d and Additional file 5: Table S3) without cytotoxicity [16].

### SHL decreases Ca^2+^_[c]_ levels and inhibits NFAT activation in the sensitized RBL-2H3 cells

As shown in Fig. 3e and Additional file 5: Table S3, SHL suppressed ST-stimulated NFAT activation in a concentration-dependent manner. NFAT are a family of Ca^2+^-dependent transcription factors [23]. Thus, we next investigated whether SHL inhibited the ST-induced Ca^2+^_[c]_ elevation in RBL-2H3 cells. As expected, ST challenge markedly elevated Ca^2+^_[c]_ levels in the sensitized RBL-2H3 cells, while a pretreatment with SHL significantly reduced Ca^2+^_[c]_ levels in a concentration-dependent manner (Fig. 3f and Additional file 5: Table S3), demonstrating that SHL suppressed ST-induced Th2 immunity via the regulation of the Ca^2+^-NFAT pathway.

## Discussion

As a traditional Chinese formula, SHL is not only used for the treatment of acute upper respiratory tract infection, acute bronchitis and light pneumonia caused by bacterium/viruses, but also applied to treat IgE-mediated allergy, such as bronchial asthma [17, 18], etc. Previous studies have showed that all of the components in SHL possess the anti-allergic activity [24–26], of which the effect of *Scutellaria baicalensis* on Th2 immunity has been identified [27], highly suggesting that SHL is also likely to suppress Th2 immunity.

Th cells play an important role in orchestrating adaptive immune responses. Th2 immunity controls the humoral immune response by triggering B cell differentiation and producing IgE via Th2 cytokines [28]. ST, a more sensitive antigen to rodents than ovalbumin [16], was used in our study. 4 weeks after ST immunization, the mice showed Th2-bias response. SHL significantly decreased ST-stimulated splenocyte Th2-cytokines (IL-4, IL-5, IL-10 and IL-13) production (Fig. 2a-d and Additional file 4: Table S2) without affecting Th1 cytokine IFN-γ (Fig. 2e and Additional file 4: Table S2). Simultaneously, ST-elevated serum tIgE and sIgE levels were also lowered by SHL (Fig. 1a-b and Additional file 3: Table S1), indicating that SHL indeed suppressed Th2 immunity.

In spite of representing less than 1% of peripheral blood leukocytes, BGs have become increasingly recognized as important innate immune cells [29]. On the one hand, BGs, as the effectors, are the main contributor to IgG-mediated anaphylaxis working through the release of platelet-activating factor, a highly potent proinflammatory phospholipid [30]. On the other hand, the activated BGs migrate into draining LNs and act as APCs by taking up and processing antigens. By releasing IL-4, BGs induces Th2 skewing upon peptide and hapten exposure [8–10], and they also promote Th2 polarization upon protein antigen exposure in the presence of DCs [7]. Even when the antigen-specific IgE appears, BGs that migrate into the LNs can still amplify the ongoing Th2 response by releasing IL-4 in greater amounts [10]. Our data showed that SHL suppressed BG activation marker expression (Fig. 3b-c and Additional file 5: Table S3). Moreover, SHL concentration-dependently decreased BG IL-4 production in the absence/presence of sIgE (Fig. 3a and d, and Additional file 5: Table S3), demonstrating that SHL inhibited BG activation.

The transcription of IL-4 is regulated by Ca^2+^-dependent transcription factors NFAT. A rise in Ca^2+^_[c]_ catalyzes the dephosphorylation of NFAT, and dephosphorylated NFAT translocates to the nucleus and subsequently initiating the transcription of IL-4 [23]. SHL significantly inhibited ST-stimulated NFAT activation in a concentration-dependent manner (Fig. 3e and Additional file 5: Table S3). Based on our recent finding that SHL rapidly decreases Ca^2+^_[c]_ levels by activating mitochondrial calcium uniporter [16], we presumed that SHL lowers BG Ca^2+^_[c]_ levels to inhibit NFAT nuclear translocation. Indeed, SHL markedly prevented the ST-induced Ca^2+^_[c]_ elevation in the anti-ST IgE-sensitized RBL-2H3 cells (Fig. 3f and Additional file 5: Table S3).

## Conclusions

**Fig. 4:**
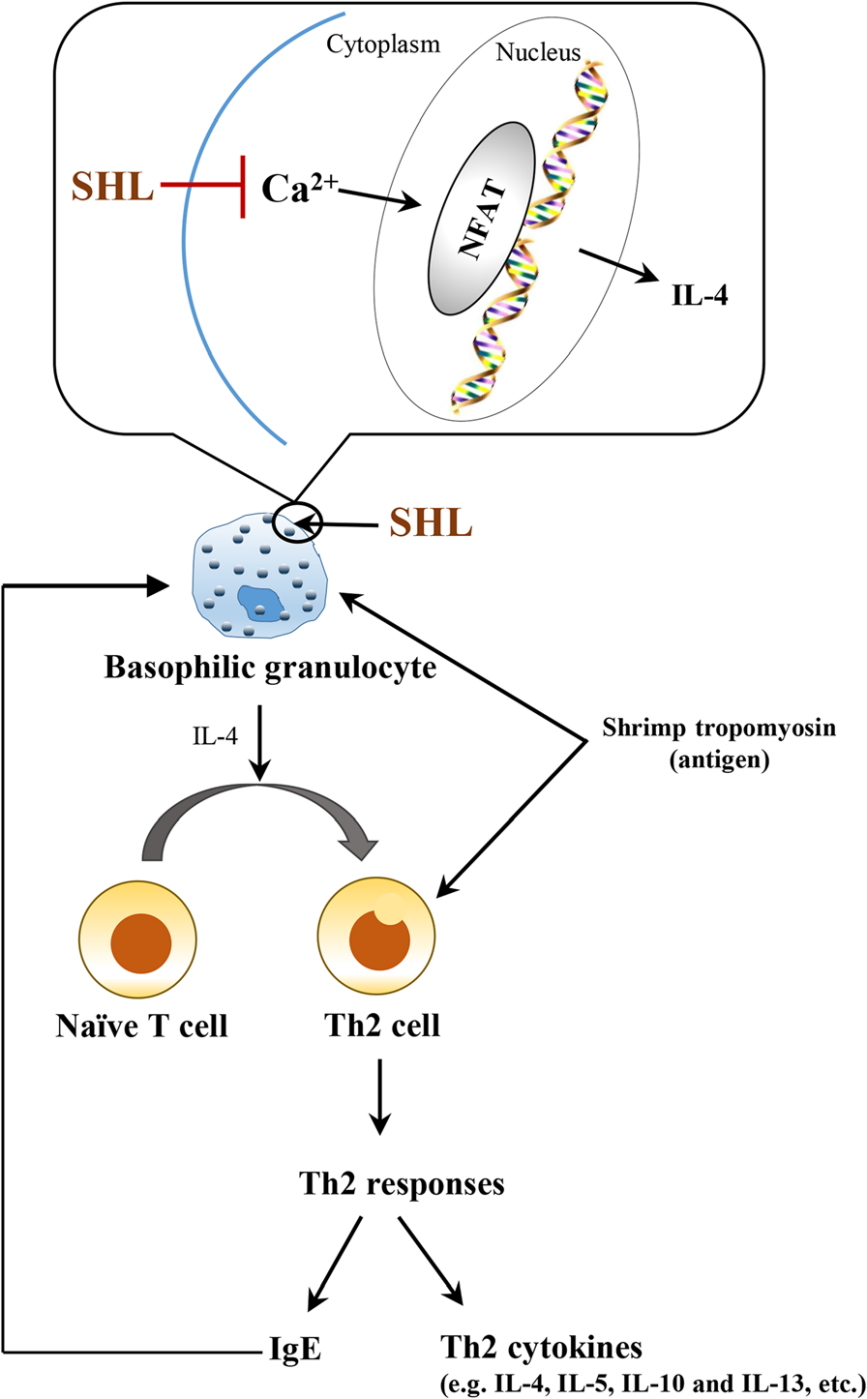
Schematic diagram depicting how SHL reduces ST-induced Th2 immunity

In summary, our findings reveal, for the first time, that SHL attenuates ST-induced Th2-cytokines release (e.g. IL-4, IL-5, IL-10 and IL-13) and serum sIgE production. This suppressive effect of SHL on Th2 immunity is attributed to its inhibition of BG activation, including suppressing CD200R surface expression and decreasing IL-4 production, in the early or middle/late stage. The latter mechanism probably involves in lowering Ca^2+^_[c]_ levels and then suppressing dephosphorylation of NFAT (Fig. 4). Our observations provide a pharmacological basis for the clinical use of SHL to relieve type I hypersensitivity by a successive dose regimen.

## Additional files

Additional file 1: Figure S1. Schedule for the preparation of the ST-sensitized mice. (DOCX 115 kb)

Additional file 2: Figure S2. Proportion of basophils in the splenocytes separated by a MACS system using a FACSCalibur flow cytometer. (DOCX 174 kb)

Additional file 3: Table S1. Raw data for Fig. 1. (DOCX 18 kb)

Additional file 4: Table S2. Raw data for Fig. 2. (DOCX 18 kb)

Additional file 5: Table S3. Raw data for Fig. 3. (DOCX 21 kb)

Additional file 6: Figure S3. Effect of SHL on the viability of basophil-rich splenocytes. The cells were treated with SHL at the indicated concentrations for 24 h. Cell viability was assessed using an MTS assay. (DOCX 84 kb)

## Abbreviations

APC: Antigen-presenting cell
BG: Basophilic granulocyte
Ca^2+^_[c]_: Cytosolic Ca^2+^
DCs: Dendritic cells
LNs: Lymph nodes
mAb: Monoclonal antibody
NCT: Naïve CD4+ T
NFAT: Nuclear factor of activated T cells
OD: Optical density
SHL: Shuang-Huang-Lian
sIgE: Specific IgE
sIgG: Specific IgG
ST: Shrimp tropomyosin
Th: T-helper
TIgE: Total IgE
