# Supplementary figures and images for: Shuang-Huang-Lian prevents basophilic granulocyte activation to suppress Th2 immunity
Source: BMC Complement Altern Med. 2018 Jan 3;18:2. doi: 10.1186/s12906-017-2071-y (PMC5753509; doi:10.1186/s12906-017-2071-y)

**
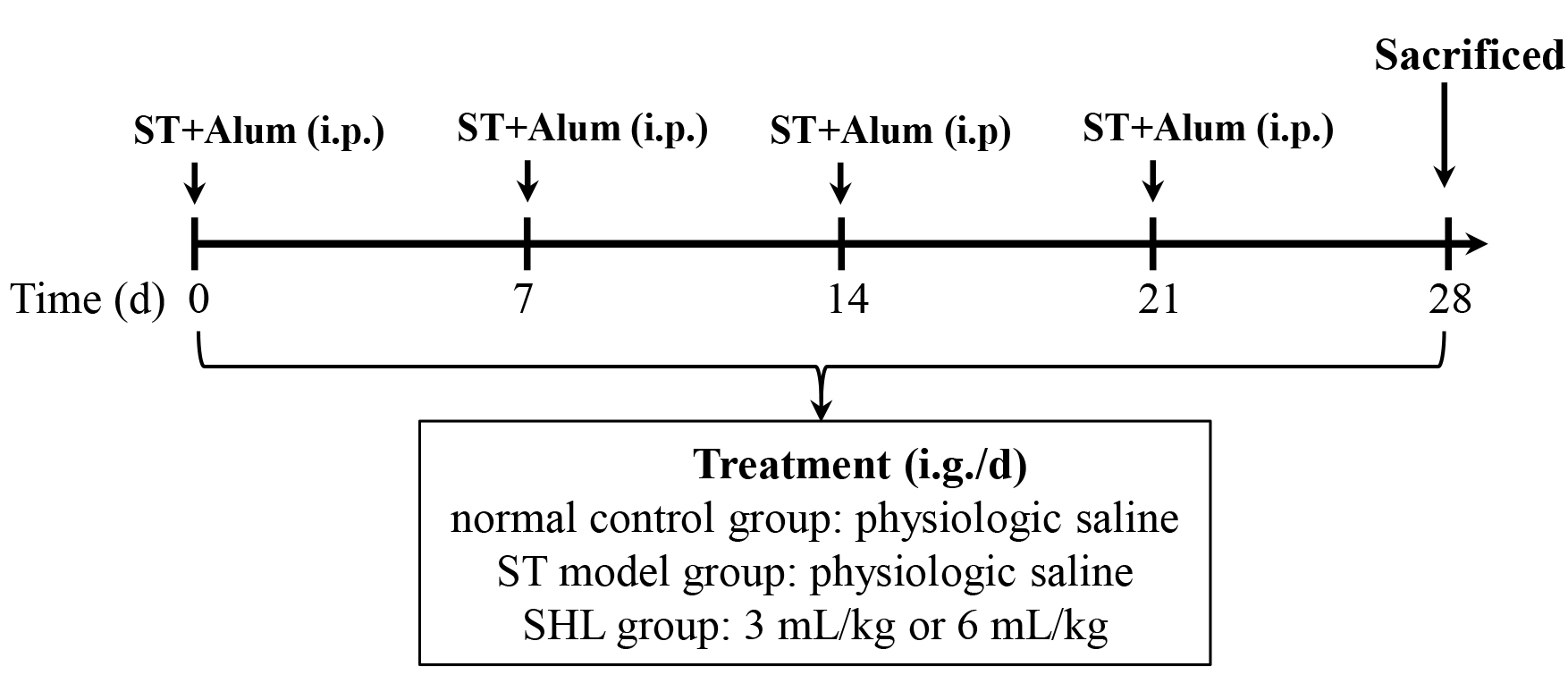
**

**Figure S1**. Schedule for the preparation of the ST-sensitized mice (8 mice per group).

Supplement: Supplementary file 1 — Schedule for the preparation of the ST-sensitized mice. (DOCX 115 kb) [file 12906_2017_2071_MOESM1_ESM.docx]
