# Supplementary material for: Shuang-Huang-Lian prevents basophilic granulocyte activation to suppress Th2 immunity
Source: BMC Complement Altern Med. 2018 Jan 3;18:2. doi: 10.1186/s12906-017-2071-y (PMC5753509; doi:10.1186/s12906-017-2071-y)
Supplement: Supplementary file 2 — Proportion of basophils in the splenocytes separated by a MACS system using a FACSCalibur flow cytometer. (DOCX 174 kb) [file 12906_2017_2071_MOESM2_ESM.docx]

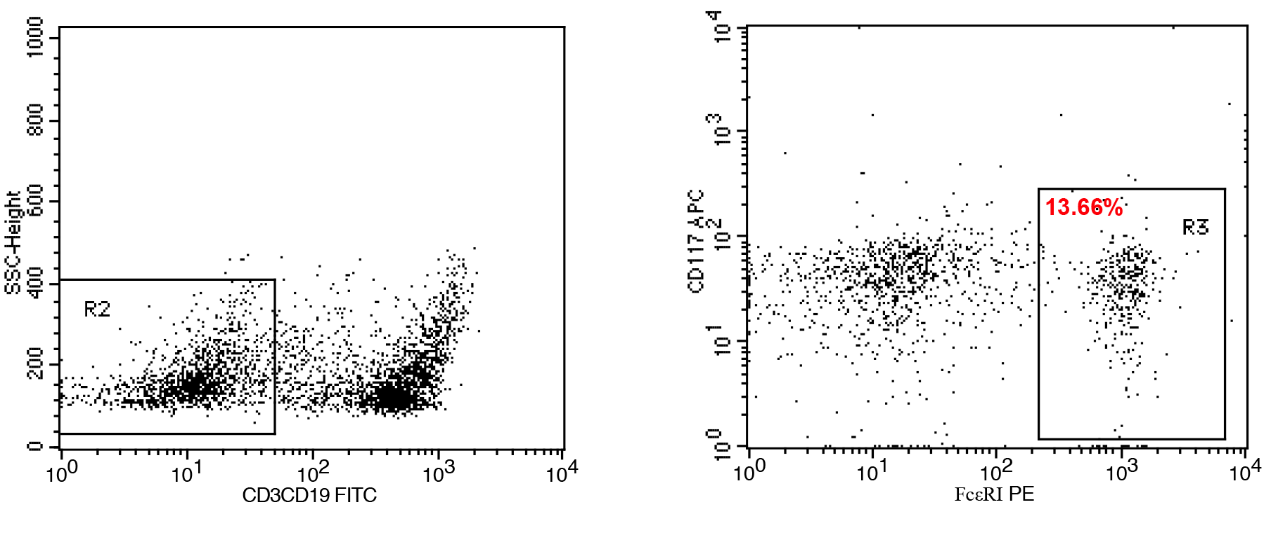


**Figure S2**. Proportion of basophils in the splenocytes separated by a MACS system using a FACSCalibur flow cytometer.
