# Supplementary material for: Shuang-Huang-Lian prevents basophilic granulocyte activation to suppress Th2 immunity
Source: BMC Complement Altern Med. 2018 Jan 3;18:2. doi: 10.1186/s12906-017-2071-y (PMC5753509; doi:10.1186/s12906-017-2071-y)
Supplement: Supplementary file 3 — Raw data for Fig. 1. (DOCX 18 kb) [file 12906_2017_2071_MOESM3_ESM.docx]

**Table S1** Raw data for figure 1.

| **tIgE** |  | |  |
| --- | --- | --- | --- |
| Group | Mean | SD | |
| Negative control | 223 | 36 | |
| ST model | 3786 | 491 | |
| SHL-3 mL/kg + ST | 2941 | 488 | |
| SHL-6 mL/kg + ST | 2940 | 384 | |
| **sIgE** |  | |  |
| Group | Mean | SD | |
| ST model | 0.559 | 0.074 | |
| SHL-3 mL/kg + ST | 0.494 | 0.061 | |
| SHL-6 mL/kg + ST | 0.471 | 0.059 | |
| **sIgG_2a_** |  | |  |
| Group | Mean | SD | |
| ST model | 0.4734 | 0.111 | |
| SHL-3 mL/kg + ST | 0.4622 | 0.053 | |
| SHL-6 mL/kg + ST | 0.4176 | 0.045 | |
| **sIgG_2b_** |  | |  |
| Group | Mean | SD | |
| ST model | 0.2233 | 0.021 | |
| SHL-3 mL/kg + ST | 0.2118 | 0.043 | |
| SHL-6 mL/kg + ST | 0.1962 | 0.037 | |
