# Supplementary material for: Shuang-Huang-Lian prevents basophilic granulocyte activation to suppress Th2 immunity
Source: BMC Complement Altern Med. 2018 Jan 3;18:2. doi: 10.1186/s12906-017-2071-y (PMC5753509; doi:10.1186/s12906-017-2071-y)
Supplement: Supplementary file 4 — Raw data for Fig. 2. (DOCX 18 kb) [file 12906_2017_2071_MOESM4_ESM.docx]

**Table S2** Raw data for figure 2.

| **IL-4** |  | |  |
| --- | --- | --- | --- |
| Group | Mean | SD | |
| Negative control | 1.01 | 0.21 | |
| ST model | 62.95 | 23.66 | |
| SHL-3 mL/kg + ST | 32.20 | 16.28 | |
| SHL-6 mL/kg + ST | 29.51 | 15.41 | |
| **IL-5** |  | |  |
| Group | Mean | SD | |
| Negative control | 10.11 | 2.41 | |
| ST model | 425.07 | 82.93 | |
| SHL-3 mL/kg + ST | 377.61 | 125.55 | |
| SHL-6 mL/kg + ST | 318.93 | 34.29 | |
| **IL-10** |  | |  |
| Group | Mean | SD | |
| Negative control | 121.30 | 12.13 | |
| ST model | 2594.08 | 829.90 | |
| SHL-3 mL/kg + ST | 2234.32 | 751.20 | |
| SHL-6 mL/kg + ST | 1514.79 | 523.73 | |

| **IL-13** |  | |  |
| --- | --- | --- | --- |
| Group | Mean | SD | |
| Negative control | 93.38 | 9.34 | |
| ST model | 1221.67 | 204.63 | |
| SHL-3 mL/kg + ST | 1252.78 | 183.14 | |
| SHL-6 mL/kg + ST | 846.11 | 252.17 | |

| **IFN-γ** |  | |  |
| --- | --- | --- | --- |
| Group | Mean | SD | |
| Negative control | 2329.41 | 232.94 | |
| ST model | 427.76 | 91.57 | |
| SHL-3 mL/kg + ST | 392.19 | 153.37 | |
| SHL-6 mL/kg + ST | 427.76 | 174.24 | |
