# Supplementary material for: Shuang-Huang-Lian prevents basophilic granulocyte activation to suppress Th2 immunity
Source: BMC Complement Altern Med. 2018 Jan 3;18:2. doi: 10.1186/s12906-017-2071-y (PMC5753509; doi:10.1186/s12906-017-2071-y)
Supplement: Supplementary file 5 — Raw data for Fig. 3. (DOCX 21 kb) [file 12906_2017_2071_MOESM5_ESM.docx]

**Table S3** Raw data for figure 3.

**A.** SHL decreased early IL-4 produced by BG-rich splenocytes.

| Group | Mean | SD |
| --- | --- | --- |
| NS | 143.18 | 7.13 |
| papain | 1015.14 | 44.14 |
| papain + 0.5% SHL | 10.09 | 6.28 |
| papain + 1% SHL | 6.18 | 2.46 |
| papain + 2% SHL | 6.18 | 6.35 |
| ST | 1142.98 | 39.92 |
| ST + 0.5% SHL | 31.58 | 3.43 |
| ST + 1% SHL | 10.42 | 5.38 |
| ST + 2% SHL | 5.53 | 2.03 |

**D.** Effect of SHL on the MFI of CD200R in the BGs after stimulation with ST.

| Group | Mean | SD |
| --- | --- | --- |
| Negative control | 262.67 | 5.03 |
| ST model | 515.30 | 80.12 |
| SHL-3 mL/kg + ST | 406.27 | 63.21 |
| SHL-6 mL/kg + ST | 334.99 | 36.33 |

**E.** SHL decreased IL-4 release in the sensitized RBL-2H3 cells.

| Group | Mean | SD |
| --- | --- | --- |
| Negative control | 23 | 1.13 |
| ST model | 371 | 22.14 |
| 0.5% SHL + ST | 185 | 10.11 |
| 1% SHL + ST | 61 | 7.6 |
| 2% SHL + ST | 24 | 0.88 |

**F.** SHL suppressed ST-induced NFAT activation in BGs.

| Group | Mean | SD |
| --- | --- | --- |
| Negative control | 1906 | 1.14 |
| ST model | 5561 | 2.92 |
| 0.5% SHL + ST | 5660 | 2.97 |
| 1% SHL + ST | 4531 | 2.38 |
| 2% SHL + ST | 4082 | 2.14 |

**G.** SHL reduced Ca^2+^_[c]_ levels in the RBL-2H3 cells.

| Relative fluorescence intensity | | | | | | | | | | | |
| --- | --- | --- | --- | --- | --- | --- | --- | --- | --- | --- | --- |
| Group/Time (s) | 0 | 30 | 60 | 90 | 120 | 150 | 180 | 210 | 240 | 270 | 300 |
| Control | 1.00 | 1.02 | 1.03 | 1.01 | 1.02 | 1.03 | 1.03 | 1.04 | 1.02 | 1.03 | 1.01 |
| ST | 1.01 | 1.25 | 1.29 | 1.28 | 1.27 | 1.28 | 1.26 | 1.26 | 1.24 | 1.29 | 1.27 |
| 0.5%SHL+ST | 0.94 | 1.17 | 1.178 | 1.18 | 1.18 | 1.21 | 1.19 | 1.17 | 1.20 | 1.18 | 1.19 |
| 1%SHL+ST | 0.79 | 1.00 | 1.00 | 1.01 | 1.02 | 1.03 | 1.05 | 1.04 | 1.00 | 1.00 | 1.01 |
| 2%SHL+ST | 0.70 | 0.87 | 0.85 | 0.85 | 0.83 | 0.84 | 0.85 | 0.85 | 0.86 | 0.84 | 0.83 |

SD value

| Group/Time (s) | 0 | 30 | 60 | 90 | 120 | 150 | 180 | 210 | 240 | 270 | 300 |
| --- | --- | --- | --- | --- | --- | --- | --- | --- | --- | --- | --- |
| Control | 0.013 | 0.010 | 0.010 | 0.012 | 0.005 | 0.006 | 0.010 | 0.010 | 0.006 | 0.008 | 0.008 |
| ST | 0.150 | 0.019 | 0.006 | 0.003 | 0.008 | 0.010 | 0.014 | 0.027 | 0.027 | 0.014 | 0.014 |
| 0.5%SHL+ST | 0.136 | 0.007 | 0.002 | 0.016 | 0.014 | 0.019 | 0.013 | 0.012 | 0.007 | 0.006 | 0.006 |
| 1%SHL+ST | 0.117 | 0.008 | 0.011 | 0.010 | 0.019 | 0.012 | 0.032 | 0.028 | 0.008 | 0.009 | 0.009 |
| 2%SHL+ST | 0.095 | 0.011 | 0.011 | 0.008 | 0.011 | 0.005 | 0.004 | 0.009 | 0.014 | 0.007 | 0.007 |
