# Supplementary material for: Shuang-Huang-Lian prevents basophilic granulocyte activation to suppress Th2 immunity
Source: BMC Complement Altern Med. 2018 Jan 3;18:2. doi: 10.1186/s12906-017-2071-y (PMC5753509; doi:10.1186/s12906-017-2071-y)
Supplement: Supplementary file 6 — Effect of SHL on the viability of basophil-rich splenocytes. The cells were treated with SHL at the indicated concentrations for 24 h. Cell viability was assessed using an MTS assay. (DOCX 84 kb) [file 12906_2017_2071_MOESM6_ESM.docx]

**
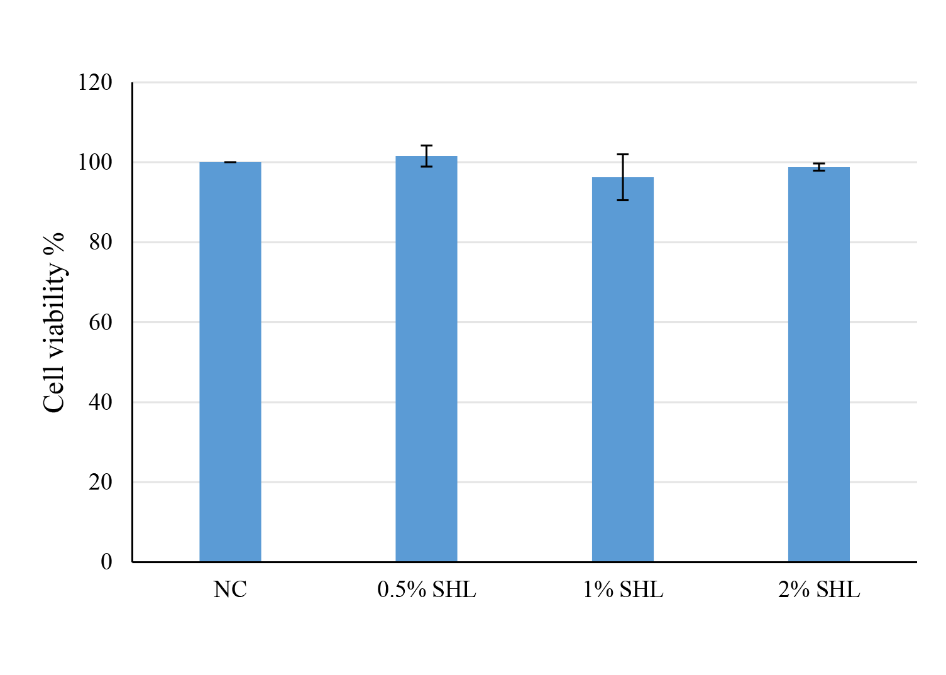
**

**Figure S3.** Effect of SHL on the viability of basophil-rich splenocytes. The cells were treated with SHL at the indicated concentrations for 24 h. Cell viability was assessed using an MTS assay.
